# Anterior approach with mini-bikini incision in open reduction in infants with developmental dysplasia of the hip

**DOI:** 10.1186/s13018-020-01700-y

**Published:** 2020-05-20

**Authors:** Guoqiang Jia, Enbo Wang, Peng Lian, Tianjing Liu, Shuyi Zhao, Qun Zhao

**Affiliations:** 1 Department of Pediatric Orthopedics, Shengjing Hospital of China Medical University, No. 36 Sanhao Street, Shenyang, China; 2 Department of Orthopedics, Anhui Provincial Children’s Hospital, Hefei, China

**Keywords:** Developmental dysplasia of the hip, Mini-bikini incision, Surgical technique, Anterior approach, Open reduction

## Abstract

**Purpose:**

The anterior and medial approaches in open reduction for developmental dysplasia of the hip (DDH) had been widely used. The former could not directly approach the intra-articular interposition, while the latter had been associated with injury to blood vessel and avascular necrosis (AVN) of the femoral head. Meanwhile, the bikini incision had also been mentioned in some studies. The purpose of this study was to introduce a modified anterior approach through a mini-bikini incision and report its short-term outcomes.

**Methods:**

Data of DDH patients younger than 2 years at the time of surgery who had received this mini-bikini incision between June 2013 and December 2018 were collected. The surgical technique, operation duration, intraoperative blood loss, and length of incision were recorded in detail. In the latest follow up, the objective measurement of the scar and the subjective feeling towards the scar were collected. X-ray and magnetic resonance imaging (MRI) were performed at the last follow-up, and the incidence of residual dysplasia, redislocation, and femoral head AVN was analyzed.

**Results:**

Forty-three cases (49 hips) were included with an averaged follow-up of 43 months. The operation duration was 22 min, and the blood loss was 9.8 ml on average. The length of the scar averaged 2.6 cm. The mean University of North Carolina “4P” scar scale (UNC4P) for the scar was 0.92, and no patients complained numbness. Overall, all the parents were satisfied with the cosmetic appearance. The mean acetabular index (AI) was 27.42° ± 6.41° in dislocated hip in the last follow-up. One hip redislocated soon after the operation and was reduced in a closed manner right away. MRI showed improved coverage but still some residual dysplasia that was in accordance with the post-operative recovery nature. Four hips (8%) had signs of AVN in X-ray.

**Conclusion:**

Open reduction through the anterior approach with the mini-bikini incision was a safe procedure with comparable outcomes to classical approaches. It would be a complementary approach for DDH patients younger than 2 years old who need an open reduction.

## Introduction

Developmental dysplasia of the hip refers to a spectrum of hip deformities, ranging from mild dysplasia to frank dislocation. In early-presenting infants, a Pavlik harness may solve the problem [1, 2]. For those who failed the treatment or the early diagnosis, closed reduction (CR) or open reduction (OR) might be necessary within 24 months of age [3–6].

OR can be achieved via an anterior or a medial approach [7–13]. The anterior approach directly approaches the contracted hip capsule and intra-articular obstacles but may leave cosmetic concerns and the risk of lateral femoral cutaneous nerve dysesthesia [7–9]. The medial approach, while facilitating the removal of the interposition, has been reported to increase the incidence of AVN and not facilitate capsulorrhaphy [10–13]. In order to address these concerns, some surgeons adopted the bikini incision and performed the Smith-Peterson (SP) approach underneath [14, 15]. This had been reported to achieve comparable outcome with, if no better than, the traditional approaches.

This study describes a modification to the bikini-SP approach. The surgical technique will be described in details and its short-term outcomes will be reported.

## Materials and methods

The study had been approved by our institution’s review board. This anterior approach with mini-bikini incision had been performed by one single senior surgeon (EW) ever since June 2013. This study retrospectively analyzed the data of patients that underwent this approach between June 2013 and December 2018. The inclusion criteria were as follows: (1) CR failed according to Bowen’s criteria [16], (2) the patients received an OR through the anterior approach with mini-bikini incision, and (3) the patients were within 2 years of age. The exclusion criteria were as follows: (1) the clinical follow-up was less than 1 year; (2) traumatic, teratogenic dislocation or with other musculoskeletal disorders; and (3) the patients received concurrent peri-acetabular or femoral osteotomy. The Bowen’s criteria were as follows: (1) the corner of the proximal femoral metaphysis was located inferior to the Hilgenreiner line, (2) the medialization ratio was greater than two thirds of the horizontal radius of the femoral head, and (3) the femoral head should maintain under the hypertrophic labrum [16].

### Surgical technique

After general anesthesia, the patient lay in the supine position on a standard operating table. Arthrography-assisted CR was attempted at first. Arthrography was performed via a medial approach with an average of 1.2 ml (0.8–1.5) iopromide (Bayer Pharmaceuticals Co, Ltd. Guangzhou Branch, Guangzhou). After closed reduction, firstly, the Bowen criteria were used to assess the result of reduction [16]. Secondly, the arc of stability in flexion/extension and abduction/adduction was evaluated and a safe zone angle < 30° was unacceptable [17]. OR was indicated if either one of the two criteria was not met.

**Fig. 1.**
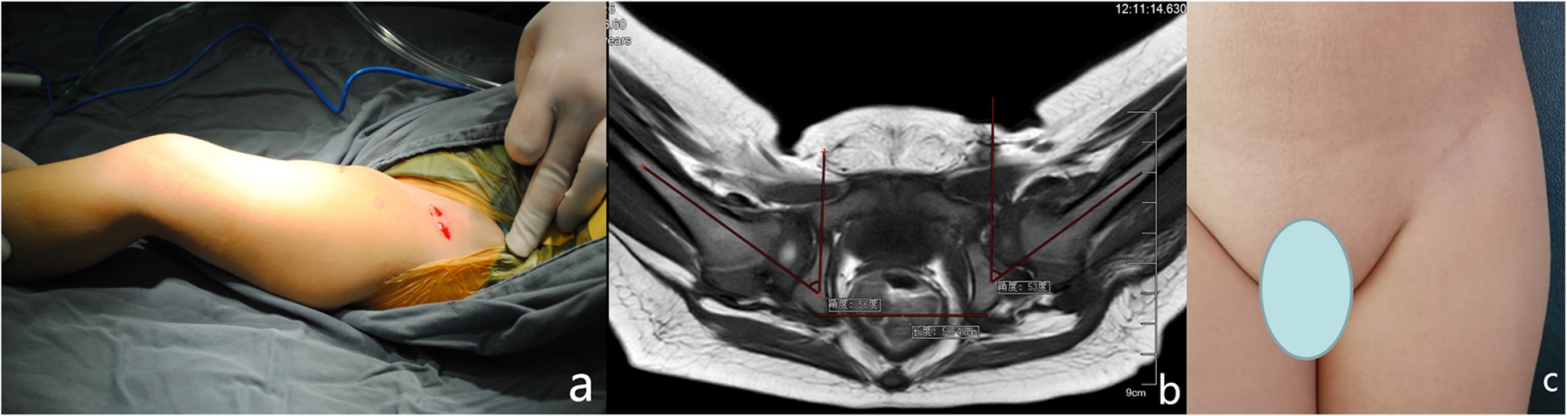
**a** The incision length was 2.5 cm along the groin crease beneath the ASIS. **b** MRI confirmed the reduction of the left femoral head. The left hip was in 53° of abduction. **c** The scar of the incision was almost invisible at 5.5 years after surgery

**Fig. 2.**
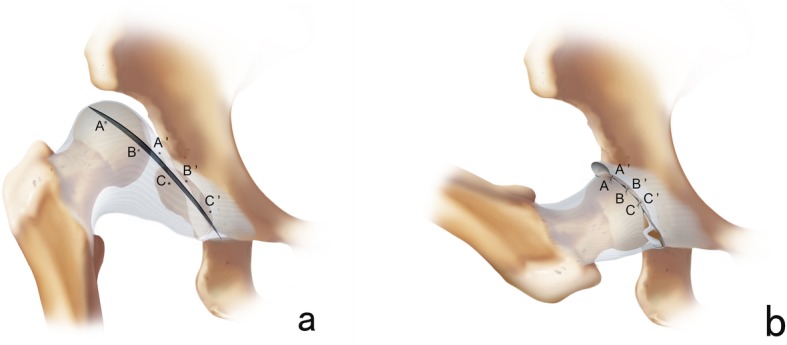
**a** Schematic diagram of the hip joint capsule incision before reduction. **b** Schematic diagram of the translational tighten sutures as the capsulorrhaphy after reduction

The incision started approximately 1 cm medial and distal to the anterosuperior iliac spine along the groin crease, and the length was 2 to 3 cm (Fig. 1a). The subcutaneous tissue was dissected transversely till the deep fascia and then went on across the medial edge of the sartorius muscle longitudinally through the interval between the iliopsoas and sartorius. The lateral femoral cutaneous nerve was recognized and carefully retracted laterally. The proximate rectus femoris was dissociated and retracted laterally to expose the anterior part of the capsule, and then an oblique incision was made instead of the traditional T-shaped incision. After releasing the iliopsoas tendon and the transverse ligament of the acetabulum, as well as excising the ligamentum teres and fibrofatty pulvinar, a capsulorrhaphy was performed by translational tighten sutures (Fig. 2). The safe zone angle was generally improved to above 40° afterward. Then, the incision would be closed cautiously and the incision would be glued up by histoacryl (Histoacryl Tissue Adhesive, Braun, Spain) without sutures. The adductor longus would be released percutaneously if necessary.

After the procedure, the hips were maintained in a spica cast in human position with 100–110° of flexion and 55° (47–62°) of abduction (Fig. 1b). The results were confirmed with an immediate postoperative MRI (Fig. 1b). The fixation time was 6–10 weeks in spica cast; after that, an abduction brace would be used 23 h a day for another 12 weeks.

### Clinical outcomes

The scar was described and recorded using the UNC4P scale, which included four aspects: pruritus, paraesthesia, pliability, and pain (0, none; 1, mild; 2, moderate; 3, severe) [18]. The patients’ feeling towards the cosmetic appearance (very satisfied, satisfied, unsatisfied, very unsatisfied) and the existence of numbness (yes, no) was collected from their parents [9]. Clinically, ranges of the passive motion were assessed and recorded.

### Image measurements

The International Hip Dysplasia Institute (IHDI) criteria were used to grade the dislocations on preoperative radiographs. The AI was measured on standard anteroposterior radiograph. Series MRI were performed before operation and regularly in follow-ups [19–21]. Cartilaginous acetabular index (CAI), cartilage coronal acetabular head index (CCAHI), and cartilage sagittal acetabular head index (CSAHI) were measured on the T1-W SE anatomic sequence in the coronal and sagittal planes that showed the largest diameter of the femoral head according to Douira’s study [22]. AVN was assessed in the last follow-up according to the Salter criteria [23].

### Statistical analysis

Statistical analyses were performed with SPSS version 22.0 (IBM Corp, Armonk, NY, USA). Data were shown as mean ± standard deviation. Paired sample *t* test was used to compare the parameters between the affected and unaffected side. Bilateral involvements were not included in this comparison. The significance level was *p* < 0.05.

## Results

A total of 50 patients met the inclusion criteria. Five lost to follow-up and two had incomplete records, leaving 43 patients with 49 hips affected in the study (Table 1). There were 37 girls and 6 boys, the mean age was 11 months at surgery, and the mean follow-up was 43 months.

**Table 1:** Demographic characteristics of the subjects

Characteristic	Total 43 patients (49 hips)
Female gender, *n* (%)	37 (86)
Side, *n* (%)	
Left to right to bilateral	22 (51):15 (35):6 (14)
IHDI, *n* (%)	
II to III to IV	1 (2): 12 (24): 36 (74)
Mean age at surgery, months (range)	11 (3–22)
Mean surgical time, min (range)	22 (18–37)
Mean follow-up time, months (range)	43 (14–75)
Mean length of incision, cm (range)	2.6 (2–3)
Mean intraoperative blood loss, ml (range)	9.8 (5–22)
Mean abduction angle in spica MRI, ° (range)	55 (47–62)
Mean follow-up age, months (range)	50 (22–74)

### Clinical outcomes

The mean abduction angle of the affected hip was 55° (47–62°) after OR in spica cast. None of the patients had limited hip movement, neither in the affected nor in the healthy side. Leg length discrepancy did not present in any of the patients. The mean UNC4P total score was 0.92, and no patients had numbness. All the parents were satisfied or very satisfied with the post-operative appearance (Fig. 1c).

### Radiological outcomes

In the latest follow-up, the mean AI was 27.42° ± 6.41°, with significant difference from the contralateral side. Cartilaginous coverage, as shown in MRI, remained inferior to the healthy side despite the gradual improvement after treatment. All the measurement of the latest follow-up was listed in Table 2.

**Table 2:** Comparison of the parameters in X-ray and MRI between the affected and unaffected sides

	Dislocated side		Contralateral side			
	Mean	SD	Mean	SD	*t* value	*p* value
CAI (°)	15.04	4.38	10.55	4.58	6.83	0.000
CCAHI (%)	77.89	7.27	82.49	6.11	2.78	0.008
CSAHI (%)	92.81	6.15	95.71	3.57	2.42	0.021
AHI (°)	27.42	6.41	21.08	4.59	5.29	0.000

Data were shown as mean ± standard deviation. *p* represented the comparison between the affected and unaffected sides of unilateral DDH cases. Bilateral cases were excluded

### Complications

**Fig. 3:**
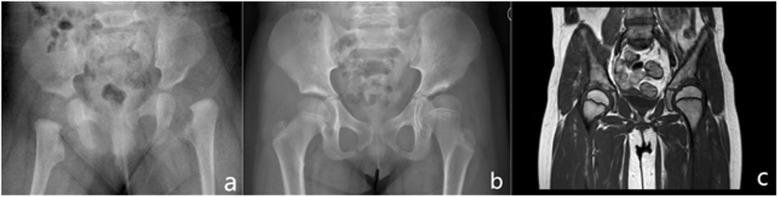
**a** Pre-operative anteroposterior radiograph of a 4-month-old girl with IHDI type III dislocation of the left hip. **b** 5.5 years after surgery, there were signs of acetabular dysplasia in the left hip on X-ray. **c** MRI showed sufficient cartilage acetabular coverage in the coronal plane

There was no deep or superficial infection in any of the patients. Most of the hips developed well with time (Fig. 3). One hip (2%) redislocated as shown by postoperative MRI, and a closed reduction was immediately performed with success. Four hips (8%) showed signs of AVN, including three boys and one girl. All of the AVN cases were IHDI type IV. The abduction angle of the affected side with AVN in spica cast were 54°, 57° ,59°, and 60°, respectively. Residual dysplasia were present in 32/49 cases according to the CAI and 28/49 cases according to the CCAHI in the coronal plane; CSAHI averaged 92.81% indicating the prevalence of mild dysplasia in the sagittal plane [22].

## Discussion

For DDH patients who failed CR before age 2, OR was usually performed. Generally, OR could be performed through the anterior or medial approach. The anterior approach for simple OR was in fact part of the classic SP approach. It provided wide and safe exposure of the joint that significantly facilitated the operation, but it was difficult to expose the inner side of the acetabulum and might cause confusion between the real and false acetabulum. By lengthening the incision, a pelvic or femoral osteotomy could be performed when necessary. The medial approach, on the contrary, provided direct vision of the intra-articular structures through a cosmetically insidious incision. However, this approach put the medial circumflex vessels at risk and was often suspected to cause more AVN than the anterior approach. Besides, when simple OR cannot achieve a stable reduction, another incision must be made to perform osteotomies.

Some surgeons adopted the bikini incision with the SP approach underneath. Since the incision was along the grease fold, it was almost invisible and left minor cosmetic concerns. Previous reports showed that the success rate and incidence of complications were comparable with traditional anterior or medial approaches [11, 13, 23–25]. This approach had also been used in total hip arthroplasty, also resulting in comparable outcomes but less scarring [9]. Our study quantified the content of scarring and focused on the patients/parents feeling about the incision. The average length of the scar was 2.6 cm. Since most previous reports did not include data about the length of incision, we cannot make comparisons with them. One study described that they made an incision of 5 cm long in the operation [14], but it was hard to make direct comparison due to the heterogeneity of the patients. Nonetheless, this mini-bikini incision was significantly shorter than our previous operations through the longitudinal anterior approach. Because the tension around the incision was minimal in the position of hip flexion, glues could be used instead of sutures. The scarring was less even when the length was the same. In this case, the subjective feeling of the patients had been greatly improved.

As to the operative techniques, we selected the muscle interval between iliopsoas and sartorius mainly because it facilitated to approach the acetabulum and release the iliopsoas tendon. We did not dissect the femoris rectus as reported previously. Instead, we retracted the tendons and muscles laterally and entered the capsule from between. In this case, we diminished the injury to surrounding tissue, reduced blood loss, shortened the operation time, and maintained the integrity of the femoris rectus. We would free the origin of the femoris rectus if it was extremely tight to reduce the femoral head. Hopefully, this would help maintain the power of the femoris rectus. Besides, the capsulotomy was achieved through an oblique incision followed by translational tightening suture. This helped simplify the traditional T-shaped incision and further shortened the operation time. Crucial steps of OR, including the clearance of intra- and extra-articular obstacles, the release of the iliopsoas tendon, the contracted capsule, and the transverse ligament, as well as the clearance of the ligamentum teres and the hypertrophic tissue in the acetabulum, could all be accomplished through this single approach. Although it seemed difficult to access the capsule without dissecting the femoris rectus, an experienced surgeon with adequate training on hip surgeries can achieve this without difficulty. Besides, for cases that needed further treatment like pelvic osteotomy, this scar would be on the track of the new incision and therefore would not add any cosmetic concern.

This approach yielded comparable short-term outcomes with similar reports [11, 13]. None of the patients had any limited hip motion, and the gait of the patients was largely normal. More than half of the patients presented with signs of hip dysplasia in MRI, with the CSAHI averaged 92.8% [22]. However, retardation in hip development would always happen in DDH hips, even in the unaffected side [26]. Since the potential for spontaneous resolution could still expected, simple follow-ups were recommended to all the patients [27]. The AVN rate was 8%, which was significantly lower than previously reported [10–13, 23–25], but the follow-up time was too short to draw any definite conclusion. All of the four AVN patients were IHDI type IV, and three were male, indicating a possible association between severe dislocation as well as male gender and the incidence of AVN. One right hip in our series re-dislocated posteriorly shortly after the OR and was successfully reduced by close reduction. Redislocation might be attributed to technical pitfalls such as inadequate release of the iliopsoas tendon or the inferomedial capsule [25] or poor casting technique. After one and a half year follow-up, this patient had 12°of CAI, 84% of CCAHI, and 96% of CSAHI, without AVN or limitation of movement.

This study has some limitations. Firstly, the follow-up of this study was short and the sample size was limited. We would continue following them up while adding new subjects to this cohort. Secondly, it was a retrospective study without randomization and controls. Therefore, the bias in selecting those patients as recipients of our novel incision might cause some deviation in the results. Lastly, due to the young age of the patients, our functional evaluation included only the passive range of motion. More sophisticated functional evaluation of the lower limbs, especially the strength of the femoris rectus, should be added to this follow-up regimen.

## Conclusions

This study introduced a modification to the bikini incision in open reduction of DDH patients younger than 2 years. It is a safe approach which would yield comparable short-term outcome as the traditional approaches, with short operation time, reduced blood loss, better muscle protection, and less scarring. This procedure could be a complementary treatment for DDH patients who failed an initial closed reduction.

## Data Availability

The datasets are available from the corresponding author on reasonable request.

## Abbreviations

DDH: Developmental dysplasia of the hip
AVN: Avascular necrosis
MRI: Magnetic resonance imaging
UNC4P: University of North Carolina “4P”
CR: Closed reduction
OR: Open reduction
SP: Smith-Peterson
IHDI: International Hip Dysplasia Institute
AI: Acetabular index
CAI: Cartilaginous acetabular index
CCAHI: Cartilage coronal acetabular head index;
CCSHI: Cartilage sagittal acetabular head index
SD: Standard deviation

## References

[1] Hines AC, Neal DC, Beckwith T, Jo C, Kim H (2019). A comparison of Pavlik harness treatment regimens for dislocated but reducible (Ortolani+) hips in infantile developmental dysplasia of the hip. J Pediatr Orthop.

[2] Mulpuri K, Song KM (2015). AAOS Clinical Practice Guideline: Detection and nonoperative management of pediatric developmental dysplasia of the hip in infants up to six months of age. J Am Acad Orthop Surg.

[3] Sankar WN, Gornitzky AL, Clarke N (2019). Closed reduction for developmental dysplasia of the hip: early-term results from a prospective, multicenter cohort. J Pediatr Orthop.

[4] Tennant SJ, Eastwood DM, Calder P, Hashemi-Nejad A, Catterall A (2016). A protocol for the use of closed reduction in children with developmental dysplasia of the hip incorporating open psoas and adductor releases and a short-leg cast: mid-term outcomes in 113 hips. Bone Joint J.

[5] Talathi Nakul S., Trionfo Arianna, Patel Neeraj M., Upasani Vidyadhar V., Matheney Travis, Mulpuri Kishore, Sankar Wudbhav N. (2020). Should I Plan to Open? Predicting the Need for Open Reduction in the Treatment of Developmental Dysplasia of the Hip. Journal of Pediatric Orthopaedics.

[6] Bulut M, Gürger M, Belhan O, Batur OC, Celik S, Karakurt L (2013). Management of developmental dysplasia of the hip in less than 24 months old children. Indian J Orthop.

[7] Morcuende JA, Meyer MD, Dolan LA, Weinstein SL (1997). Long-term outcome after open reduction through an anteromedial approach for congenital dislocation of the hip. J Bone Joint Surg Am.

[8] Rudin D, Manestar M, Ullrich O, Erhardt J, Grob K (2016). The anatomical course of the lateral femoral cutaneous nerve with special attention to the anterior approach to the hip joint. J Bone Joint Surg Am.

[9] Leunig M, Hutmacher JE, Ricciardi BF, Impellizzeri FM, Rüdiger HA, Naal FD (2018). Skin crease ‘bikini’ incision for the direct anterior approach in total hip arthroplasty. A two- to four-year comparative study in 964 patients. Bone Joint J.

[10] Kalamchi A, Schmidt TL, MacEwen GD (1982). Congenital dislocation of the hip: open reduction by the medial approach. Clin Orthop Relat Res.

[11] Hoellwarth JS, Kim YJ, Millis MB, Kasser JR, Zurakowski D, Matheney TH (2016). Medial versus anterior open reduction for developmental hip dislocation in age-matched patients. J Pediatr Orthop.

[12] Pollet V, Van Dijk L, Reijman M, Castelein R, Sakkers R (2018). Long-term outcomes following the medial approach for open reduction of the hip in children with developmental dysplasia. Bone Joint J.

[13] Presch C, Eberhardt O, Wirth T, Fernandez FF (2019). Comparison of arthroscopic and open reduction of conservatively irreducible dislocated hips of children. J Child Orthop.

[14] Castañeda P, Masrouha KZ, Ruiz CV, Moscona-Mishy L (2018). Outcomes following open reduction for late-presenting developmental dysplasia of the hip. J Child Orthop.

[15] Szepesi K, Szücs G, Szeverényi C, Csernátony Z (2013). Long-term follow-up of DDH patients who underwent open reduction without a postoperative cast. J Pediatr Orthop B.

[16] Forlin E, Choi IH, Guille JT, Bowen JR, Glutting J (1992). Prognostic factors in congenital dislocation of the hip treated with closed reduction. The importance of arthrographic evaluation. J Bone Joint Surg Am.

[17] Ramsey PL, Lasser S, MacEwen GD (1996). Congenital dislocation of the hip: use of the Pavlik harness in the child during the first six months of life. J Bone Joint Surg Am.

[18] Hultman CS, Friedstat JS, Edkins RE, Cairns BA, Meyer AA (2014). Laser resurfacing and remodeling of hypertrophic burn scars: the results of a large, prospective, before-after cohort study, with long-term follow-up. Ann Surg.

[19] Dibello D, Odoni L, Pederiva F, Carlo DV (2019). MRI in post reduction evaluation of developmental dysplasia of the hip: our experience. J Pediatr Orthop.

[20] Dogan O, Caliskan E, Duran S, Bicimoglu A (2019). Evaluation of cartilage coverage with magnetic resonance imaging in residual dysplasia and its impact on surgical timing. Acta Orthop Traumatol Turc.

[21] Zhou Y, Ju L, Lou Y, Wang B (2019). Analysis of acetabulum in children with developmental dysplasia of the hip by MRI scan. Medicine (Baltimore).

[22] Douira-Khomsi W, Smida M, Louati H (2010). Magnetic resonance evaluation of acetabular residual dysplasia in developmental dysplasia of the hip: a preliminary study of 27 patients. J Pediatr Orthop.

[23] Salter RB, Kostuik J, Dallas S (1969). Avascular necrosis of the femoral head as a complication of treatment for congenital dislocation of the hip in young children: a clinical and experimental investigation. Can J Surg.

[24] Cordier W, Tönnis D, Kalchschmidt K, Storch KJ, Katthagen BD (2015). Long-term results after open reduction of developmental hip dislocation by an anterior approach lateral and medial of the iliopsoas muscle. J Pediatr Orthop B.

[25] Sankar WN, Young CR, Lin AG, Crow SA, Baldwin KD, Moseley CF (2011). Risk factors for failure after open reduction for DDH: a matched cohort analysis. J Pediatr Orthop.

[26] Li LY, Zhang LJ, Li QW, Zhao Q, Jia JY, Huang T (2012). Development of the osseous and cartilaginous acetabular index in normal children and those with developmental dysplasia of the hip: a cross-sectional study using MRI. J Bone Joint Surg (Br).

[27] Li YiQiang, Liu Hang, Guo YueMing, Xu HongWen, Xun FuXing, Liu YanHan, Yuan Zhe, Li JingChun, Pereira Bruno, Canavese Federico (2020). Variables influencing the pelvic radiological evaluation in children with developmental dysplasia of the hip managed by closed reduction: a multicentre investigation. International Orthopaedics.

